# Carbohydrate handling in frugivorous tambaqui *(Colossoma macropomum)*: enzymatic and glycemic responses to dietary sugars

**DOI:** 10.1007/s00360-026-01671-2

**Published:** 2026-06-27

**Authors:** Gabriela Castellani Carli, Thaise Mota Sátiro, Douglas de Souza Graciano, Maria Karolaine Moriman Delgado, Affonso Gama Souza Pinheiro, Cristiele da Silva Ribeiro, Leonardo Susumu Takahashi

**Affiliations:** 1 Aquaculture Center of UNESP, São Paulo State University (UNESP), Jaboticabal Campus. Via de Ac. Prof. Paulo Donato Castellane, s/n, Jaboticabal, Brazil; 2 School of Engineering, São Paulo State University (UNESP), Ilha Solteira Campus. Avenue n. 56 - Ilha Solteira, São Paulo, 15385-007 Brazil; 3 Faculdade de Ciências Agrárias e Tecnológicas, FCAT, UNESP, Universidade Estadual Paulista, Dracena Campus, Rod. Comte. João Ribeiro de Barros, Km 651, Dracena, São Paulo, 17900-000 Brazil

**Keywords:** Non-protein energy, Tambaqui, Tolerance, Sugars

## Abstract

Tambaqui (*Colossoma macropomum*) is a fruit and seed-consuming omnivorous frugivorous fish. It has the metabolic capacity to process dietary carbohydrates, representing an interesting biological model for metabolic studies. We aimed to evaluate the glycaemic response and intermediate metabolism of tambaqui after administering different carbohydrates. A total of 300 juvenile tambaqui with an average weight of 111.5 ± 49.8 g were used. The fish were orally administered 200 mg/100 g body weight of glucose, fructose, sucrose, starch, or saline solution (control). Blood and liver samples were collected at 0 h (baseline) and 3, 6, 9, and 12 h after administration. Plasma glucose concentration, blood metabolites, and the hepatic activity of key metabolic enzymes were analysed. Elevated plasma glucose concentrations were observed 3 h after glucose, fructose, and sucrose administration and 6 h after starch administration. After 9 h, blood glucose levels in all groups returned to baseline. Increased hepatic activity of pyruvate kinase and glucokinase was observed 9 h after the administration of all the tested sugars. This was associated with a decrease in the activity of the gluconeogenic enzyme fructose-1,6-bisphosphatase. Fish fed starch and glucose exhibited increased hepatic fatty acid synthase activity at 6 and 9 h, respectively. Tambaquis have a high capacity to use all the tested sugars for energy purposes. However, fructose and sucrose did not stimulate metabolic synthesis of lipids because of excess sugar intake.

**Graphical abstract:**

**Figure:**
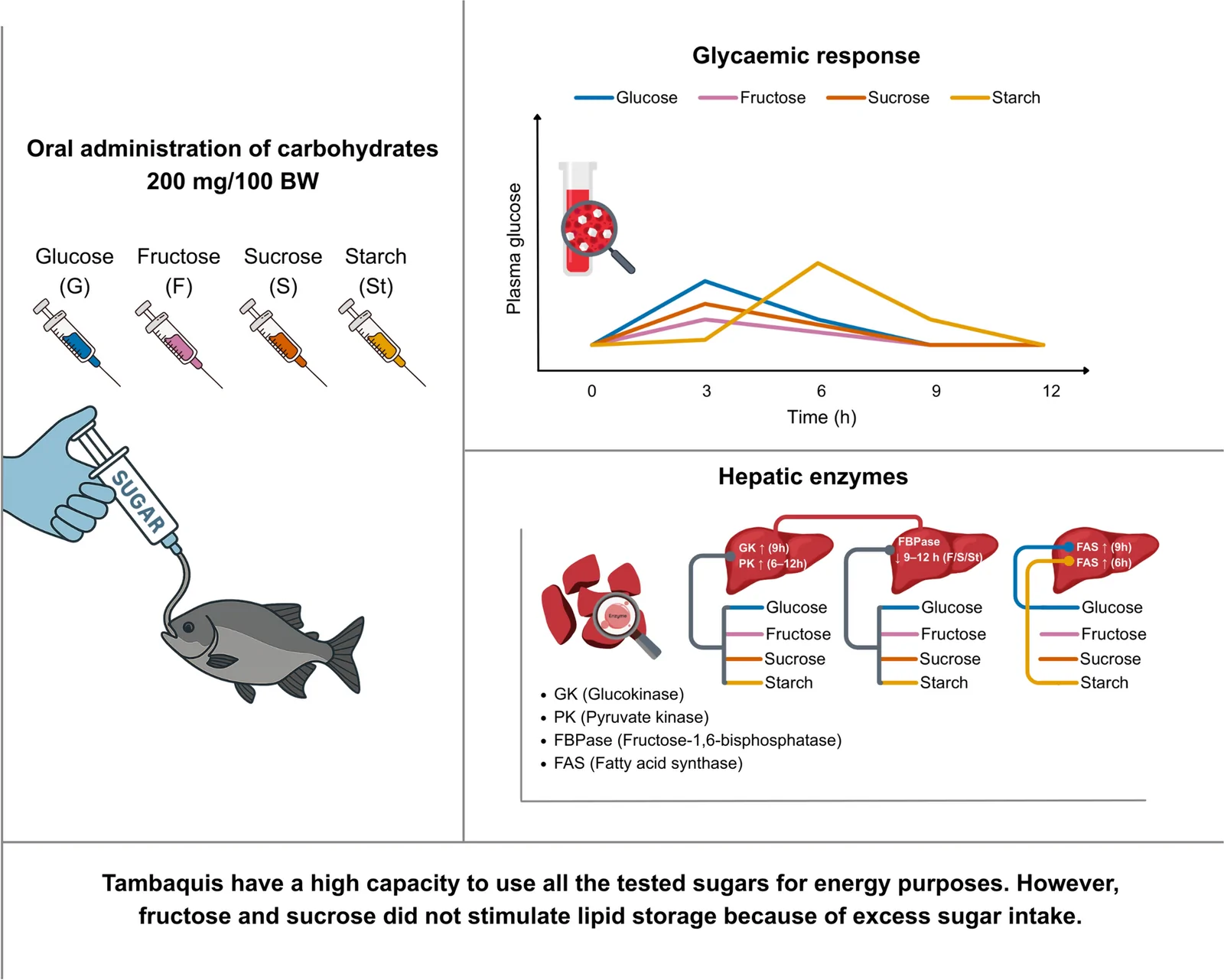

## Introduction

Carbohydrates are the most affordable energy sources. When included at appropriate levels in fish diets, they can spare dietary protein for growth and reduce nitrogen excretion (Carli et al. 2024; Liu et al. 2018), in addition to lowering feed costs. However, some species cannot efficiently metabolise dietary carbohydrates. Certain fish have been described as low-glucose tolerant, exhibiting prolonged hyperglycaemia after a glucose load. This may persist for several hours or even more than a day (Lópes-Olmeda et al. 2009; Souza et al. 2021). In the context of the present study, carbohydrate tolerance is defined operationally as the ability to regulate postprandial blood glucose levels following an acute carbohydrate challenge, as reflected by the magnitude of glycaemic peaks and the time required to restore glucose homeostasis. These effects vary depending on factors such as the species, digestive physiology, transport mechanisms, enzymatic activities, and the complexity of the carbohydrates used (Carli et al. 2024; Farias et al. 2021; Kalamalan et al. 2017; Takahashi et al. 2018).

In general, omnivorous and herbivorous fish have higher carbohydrate use rates, which are directly related to glucose uptake by the peripheral tissues (Polakof et al. 2012). Metabolic rate is likely positively correlated with hepatic glycogen and lipid accumulation in fish, functioning as an adaptive mechanism to oxidise excess dietary carbohydrates (Enes et al. 2009; Fu and Xie 2005; Kamalam et al. 2017). Therefore, herbivorous and omnivorous fish may tolerate higher carbohydrate inclusion in their diets because they naturally consume a variety of plant-based materials. Tambaqui (*Colossoma macropomum*) is an omnivorous and frugivorous species that feeds on a wide range of fruits and seeds (Hilsdorf et al. 2022), suggesting that it may efficiently use these nutrients in formulated diets.

Despite extensive knowledge on fish nutrition, specific information regarding nutrient metabolism remains limited. This study aimed to understand the adaptive metabolic characteristics of tambaqui in response to different sources of dietary carbohydrates. Oral, intraperitoneal, and intravenous glucose tolerance tests (GTTs) are fast and effective methods for estimating the glucose use capacity of animals, including fish (Liu et al. 2018; Takahashi et al. 2018). Therefore, the objective of this study was to characterize the glycaemic response curves of tambaqui following oral administration of glucose, fructose, sucrose, and starch, and to evaluate carbohydrate tolerance based on postprandial glucose regulation and associated hepatic metabolic responses.

## Material and methods

The experiment was conducted at the Aquaculture Laboratory of São Paulo State University (UNESP), Dracena Campus, São Paulo, Brazil (latitude 21°29′ S; longitude 51°32′ W).

### Fish and experimental conditions

A total of 300 tambaqui juveniles with an average weight of 111.5 ± 49.8 g were randomly distributed into 30 polyethylene tanks (200 L) at a density of 11 fish per tank, arranged in a recirculating aquaculture system equipped with a heat exchanger and biological filter. Fish were a 15-d acclimation period and fed a commercial extruded diet for omnivorous fish (28% crude protein; Presence, Santa Rosa, RS, Brazil) twice daily (9:00 am and 3:00 pm). Throughout the experimental period, the physicochemical parameters of the water remained within the recommended ranges for tambaqui farming (Hilsdorf et al. 2022). The average recorded values were temperature 28 ± 1.5 °C, dissolved oxygen concentration 7.4 ± 0.52 mg/L, and total ammonia concentration 0.25 ± 0.03 mg/L.

### Experimental procedure

Fish were fasted for 24 h to ensure clearance of the gastrointestinal tract. Six tanks were randomly assigned to each test substance. To minimise handling and stress, the first three tanks in each group were used for sample collection at 0, 3, and 9 h after administration. Meanwhile, the remaining three tanks were used for collection at 6 and 12 h. The selected sampling time points (0, 3, 6, 9, and 12 h) were defined based on previous carbohydrate tolerance studies in tambaqui (Ferrari et al. 2022) and in the closely related frugivorous species pacu (Takahashi et al. 2018), encompassing baseline conditions, the expected glycaemic peak, and the recovery phase.

Glucose, fructose, sucrose (all from Sigma-Aldrich, Merck KGaA, Darmstadt, Germany), and starch (Ingredion, São Paulo, Brazil) were all diluted in a ratio of 40 g of sugar to 100 mL of 0.9% saline solution. The control group received saline solution (0.9% NaCl, Synth, São Paulo, Brazil).

Initially, three fish per tank were sampled to determine baseline conditions (0 h). After baseline sampling, carbohydrates were administered by oral gavage at a dose of 200 mg/100 g body weight according to the method of Takahashi et al. (2018). The fish were anaesthetised in a benzocaine solution (100 mg/L), individually weighed to calculate the required volume of each sugar solution, and gavaged. Following administration, three fish per tank from each set were sampled at the specified time points (n = 9 per treatment) for biological material collection. The fish were anaesthetised again for blood sampling via caudal vein puncture and subsequently euthanised with a lethal dose of benzocaine (300 mg/L) for liver tissue collection. The liver samples were immediately stored in liquid nitrogen (− 200 °C).

To ensure that the administered solution was not regurgitated after oral gavage, preliminary trials were conducted prior to the experiment. A starch solution containing methylene blue dye was administered to a subset of fish, which were monitored for signs of regurgitation and subsequently dissected to confirm the presence of the solution in the stomach. This procedure follows the approach previously applied in pacu (*Piaractus mesopotamicus*) during oral carbohydrate tolerance tests (Takahashi et al. 2018).

### Sample processing and biochemical indicators

For plasma collection, 10 µL of anticoagulant (potassium fluoride; Glistab, Labtest Diagnóstica SA, Lagoa Santa, Brazil) was added to each microtube. For serum collection, the blood samples were incubated at room temperature (25 °C) for 1 h. Plasma and serum were separated by centrifugation (827 g, 10 min, 4 °C), and the following blood metabolites were analysed: plasma glucose (Glucose Liquiform, Labtest Diagnóstica SA, Lagoa Santa, Brazil), serum triglycerides (Triglycerides Liquiform, Labtest Diagnóstica SA, Lagoa Santa, Brazil), serum albumin (Albumin Liquiform, Labtest Diagnóstica SA, Lagoa Santa, Brazil), and total serum lipids (Frings et al. 1972).

A portion of the liver tissue was used to determine the hepatic glycogen content according to the method described by Moon et al. (1989). Another portion was homogenised (Potter NT 136, Equipamentos para Laboratórios, Piracicaba, Brazil) in ice-cold buffer [100 mM Tris–HCl (Sigma-Aldrich, Saint Louis, USA), 0.1 mM EDTA (Sigma-Aldrich, Saint Louis, USA), and 0.1% (v/v) Triton X-100 (Labsynth Produtos para Laboratórios Ltda, Diadema, Brazil), pH 7.8] at a 1:4 dilution ratio, followed by centrifugation (15 min, 25,000 g, 10 °C).

The key enzymes involved in carbohydrate metabolism were analysed: hexokinase (HK; EC 2.7.1.1) and glucokinase (GK; EC 2.7.1.2), following the methodology described by Vijayan et al. (1990), and pyruvate kinase (PK; EC 2.7.1.40), as described by Morales et al. (1990). For the lipogenesis pathway, the enzymes analysed were glucose-6-phosphate dehydrogenase (G6PDH; EC 1.1.1.49), as described by Morales et al. (2004), and fatty acid synthase (FAS; EC 2.3.1.38), as described by Chang et al. (1967). In the gluconeogenic pathway, the activity of fructose-1,6-bisphosphatase (FBPase; EC 3.1.3.11) was evaluated as described by Morales et al. (1990).

All the enzyme activities are expressed as milliunits per milligram of soluble protein (specific activity). One unit of enzymatic activity was defined as the amount of enzyme required to convert 1 µmol of substrate per minute under the specified assay conditions. Total protein concentration in the homogenate was determined using the biuret method, as described by Reinhold (1953).

### Analysis of results

The experiment was conducted using a completely randomised design arranged in a 5 × 5 factorial scheme. This design encompassed five administered substances (glucose, fructose, sucrose, starch, and saline solution) assessed across five distinct collection time points (0, 3, 6, 9, and 12 h post-administration), corresponding to 25 treatment combinations, each replicated three times. The collected data were initially evaluated for normality using the Shapiro–Wilk test and for homogeneity of variances using Bartlett’s test. Two-way analysis of variance (ANOVA) was performed, with time and sugar type as independent factors. Where significant effects were detected, post-hoc comparisons were conducted using Tukey’s honest significant difference (HSD) test at a significance level of p < 0.05. All the statistical analyses were performed using the R Development Core Team package in the RStudio software environment, version 4.4.0.

## Results

### Glycemic response of tambaqui following administration of different substances

**Fig. 1:**
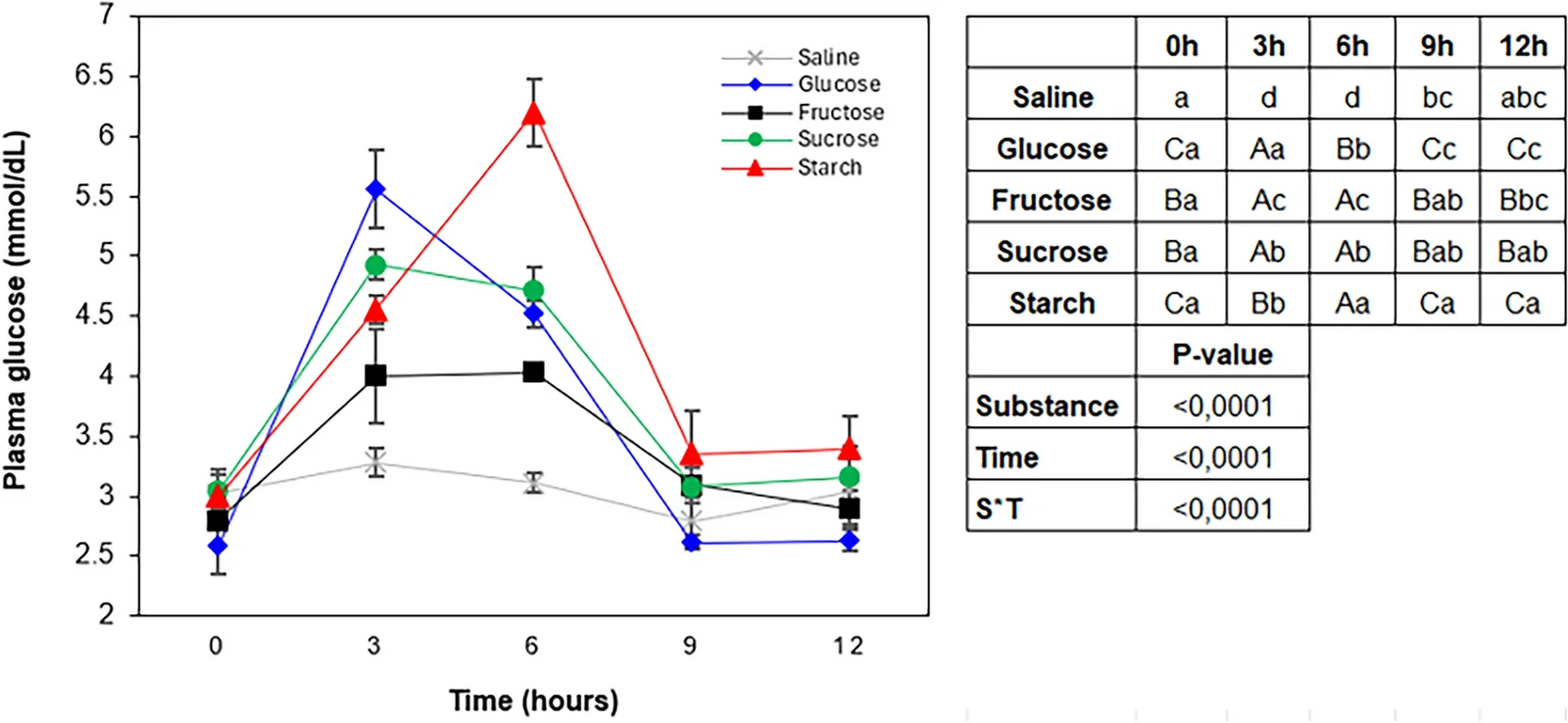
The glycemic curve of tambaquis hours after oral administration of saline solution, glucose, fructose, sucrose and starch

Significant interaction effects between the administered substances and sampling time were observed on the plasma glucose levels of tambaqui. (Fig. 1). The baseline glucose levels of the fish ranged from 2.58 to 3.04 mmol/L. Fish in the control group received saline solution and maintained glucose concentrations throughout the experiment (p > 0.05). At the 3-h interval post-administration, the highest circulating glucose concentrations among the assessed time points were evident in the groups administered with glucose (5.56 ± 0.33 mmol/L), sucrose (4.56 ± 0.33 mmol/L), and fructose (4.00 ± 0.39 mmol/L). Conversely, fish treated with starch had the highest glucose concentrations at the 6-h sampling point following administration (6.20 ± 0.28 mmol/L). By the 9-h mark, the glucose concentrations in all treatment groups returned to levels comparable to the initial baseline values.

### Parameters related to energy metabolism of tambaqui following sugar administration

**Fig. 2:**
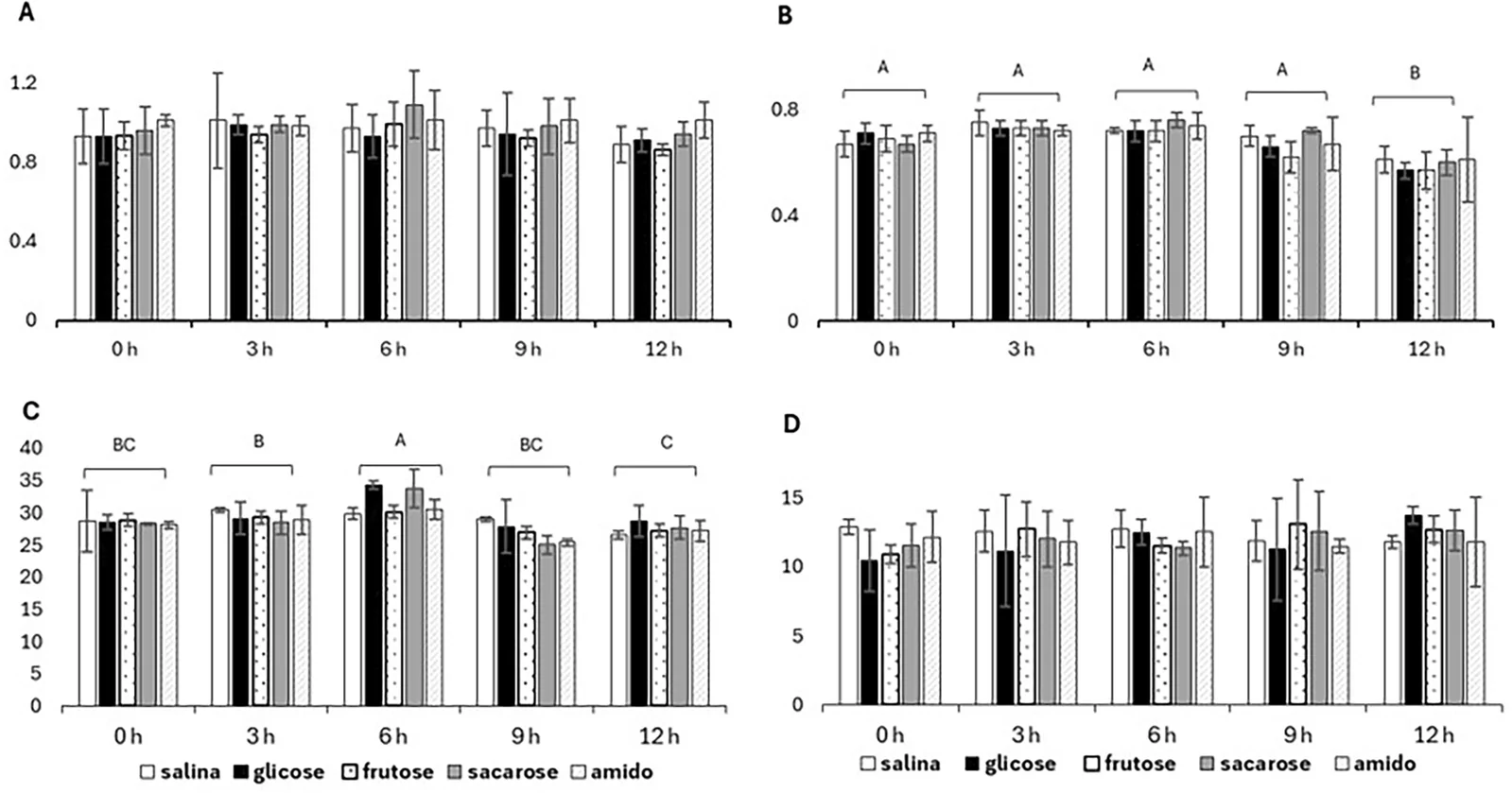
Blood metabolites and hepatic glycogen of tambaquis hours after oral administration of saline solution, glucose, fructose, sucrose and starch

Serum triglyceride levels in tambaqui specimens did not exhibit significant differences (p > 0.05) between treatments involving various administered substances, different temporal sampling points, or their interactive effects (Fig. 2A). However, serum albumin concentrations showed significant variation (p < 0.05) solely as a function of time, with a reduction observed at the 12-h sampling interval (Fig. 2B). Similarly, total serum lipid concentrations were significantly influenced by sampling time (p < 0.05), demonstrating an increase at the 6-h mark followed by a decrease at the 12-h mark across all experimental treatments (Fig. 2C). No statistically significant differences (p > 0.05) were observed in the hepatic glycogen concentrations of the tambaqui specimens following oral administration of various substances across the designated sampling time points (Fig. 2D).

### Hepatic activity of key metabolic enzymes

**Fig. 3:**
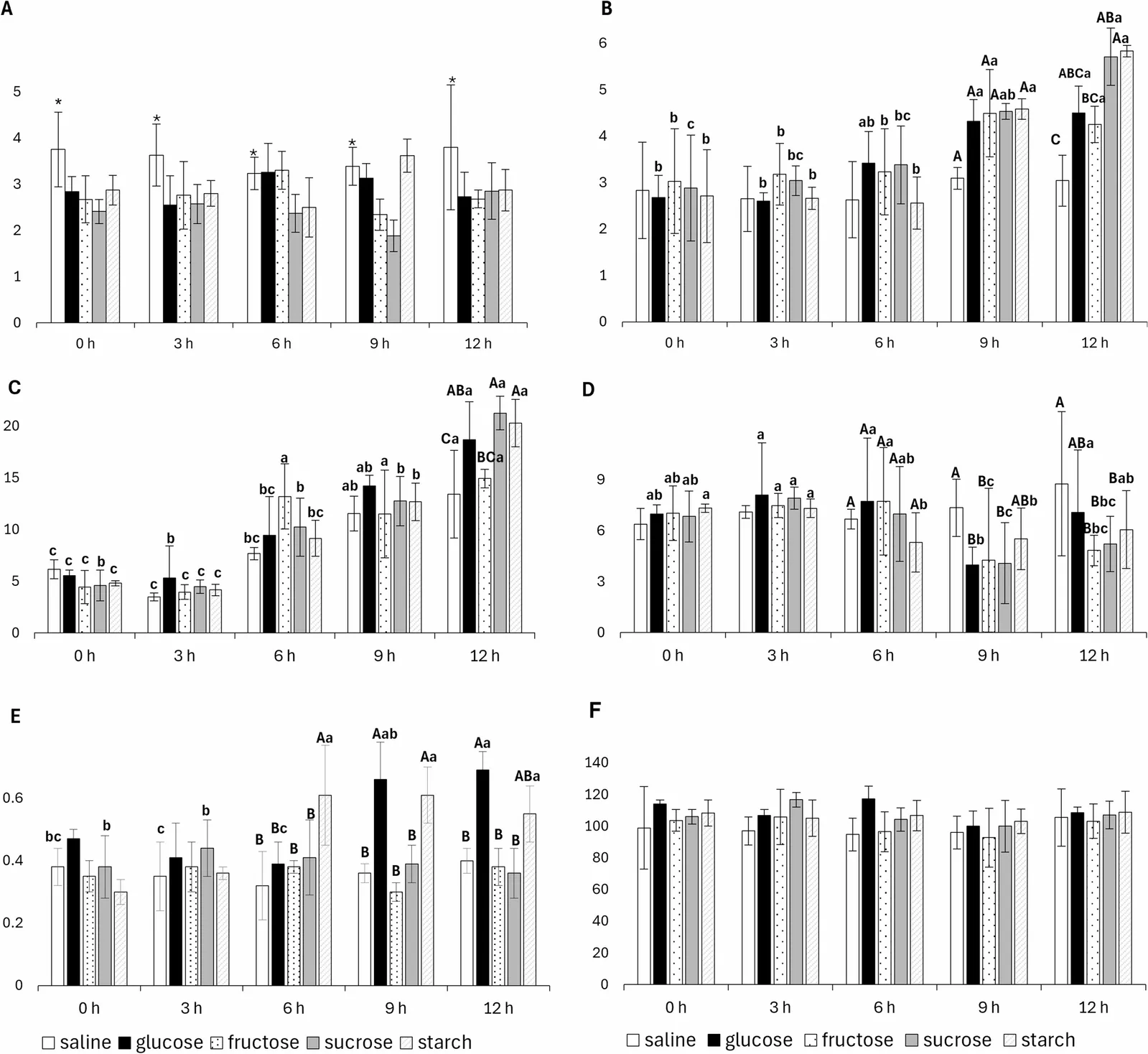
Hepatic activity of key enzymes of glycolysis, gluconeogenesis and lipogenesis of tambaquis hours after oral administration of saline solution, glucose, fructose, sucrose and starch

In the lipogenic pathway, hepatic G6PDH activity remained unchanged across the treatments (Fig. 3F). However, FAS activity showed a significant interaction between the administered substances and sampling time (S × T, p < 0.05, Fig. 3E). In the group receiving starch, the FAS activity increased starting at 6 h post-administration and remained elevated until the 12-h endpoint. Conversely, in the group treated with glucose, a significant increase in FAS activity was noted from the 9-h mark onwards. The experimental groups that received fructose, sucrose, or saline did not show any significant alterations in FAS activity during the experimental period (p > 0.05).

Regarding the glycolytic pathway, the hepatic activity of the hexokinase (HK) enzyme varied significantly only with respect to the administered substance, with statistically higher activity observed in the saline control group than in the in the other treatments (Fig. 3A). For glucokinase (GK or HK-IV), a significant interaction was observed between the administered substance and sampling time (p < 0.05, Fig. 3B). GK activity increased starting 9 h after the administration of glucose, fructose, sucrose, and starch. The control group did not show any significant temporal changes in GK activity. PK hepatic activity also demonstrated a significant interaction effect between the administered substances and sampling time (p < 0.05, Fig. 3C) Elevated activity levels were observed at 6, 9, and 12 h in the groups treated with glucose, fructose, sucrose, and starch. The control group maintained constant PK activity throughout the experimental period (Fig. 3).

Concerning the gluconeogenic pathway, the hepatic activity of fructose-1,6-bisphosphatase (FBPase) was significantly influenced by the interaction between the administered substances and the sampling times (p < 0.05) (Fig. 3D). A reduction in FBPase activity was observed at the 9-h time point following the administration of glucose, fructose, sucrose, and starch, except for the saline control group. At the 12-h mark, this reduction persisted in the fructose, sucrose, and starch treatment groups. Fish in the saline control group maintained constant FBPase activity throughout the experimental period.

## Discussion

GTTs are widely used for assessing the efficiency of carbohydrate metabolism in fish. Prolonged hyperglycaemic responses have been observed in some species. In carnivorous teleosts such as the Japanese flounder (*Paralichthys olivaceus*), peak glucose concentrations were observed 5 h after intraperitoneal (IP) administration of 1000 mg glucose per kilogram of body weight (BW), with a subsequent return to basal levels within 48 h (Liu et al. 2018). Rainbow trout had sustained hyperglycaemia for 18 h following an IP glucose load of 300 mg/kg BW (Harmon et al. 1991). Meanwhile, traira (*Hoplias malabaricus*) maintained elevated plasma glucose levels for 16 h after receiving an IP glucose load of 1000 mg/kg BW (Souza et al. 2021).

However, the temporal dynamics of hyperglycaemia are species-specific. Generally, species with a more pronounced carnivorous dietary profile require a longer time to reestablish basal glucose concentrations (Moon (2001)). In omnivorous species such as Nile tilapia (*Oreochromis niloticus*), peak plasma glucose levels were recorded 1 h after IP administration of 1000 mg glucose/kg BW, with a return to baseline within 3 h post-administration (Chen et al. 2020). Similarly, the zebrafish (*Danio rerio*) displayed a glucose peak at 0.5 h following IP injection of 1000 mg glucose/kg BW, with a return to basal levels within 6 h post-injection (Eames et al. 2010). These comparative findings suggest a potentially enhanced glucose use capacity in omnivorous fish compared to their carnivorous counterparts.

Tambaquis exhibited a robust capacity to metabolise the administered carbohydrate boluses, with peak plasma glucose concentrations observed 3 h after the oral administration of glucose, fructose, and sucrose, and 6 h after starch administration. Between 6 and 9 h post-administration, all the experimental subjects exhibited restoration of blood glucose homeostasis. Ferrari et al. (2021) reported analogous findings in tambaqui with a mean weight of 111.5 ± 49.8 g, subjected to glucose and fructose tolerance assays via IP administration. These investigators observed peak plasma glucose concentrations at 3 h post-administration of glucose and fructose, with a more rapid recovery of blood glucose homeostasis in specimens receiving IP fructose.

According to Ma et al. (2023), the absorption kinetics and metabolic rates of glucose are accelerated following intraperitoneal injection compared with oral delivery, which necessitates intestinal absorption. These authors further posited that the comparatively slower digestive processing of carbohydrates might contribute to protracted postprandial hyperglycaemia in fish. However, in our study, tambaqui returned to baseline blood glucose concentrations within 6–9 h after oral administration. Although oral and intraperitoneal routes involve distinct absorption dynamics and physiological mechanisms, the overall temporal pattern observed here falls within the same general time frame reported by Ferrari et al. (2021) in tambaqui subjected to intraperitoneal carbohydrate administration. This comparison should therefore be interpreted as indicative of broadly similar glycaemic recovery timing rather than direct physiological equivalence.

Takahashi et al. (2018) conducted oral glucose, fructose, and starch tolerance tests (gavage) on juvenile Pacu with a mean weight of 350 g. They reported elevated plasma glucose levels in fructose-administered fish 2 h post-gavage, with a return to basal levels within 4 h. Fish receiving glucose and starch exhibited peak glucose concentrations at 4 h, returning to baseline levels at 12 and 6 h post-administration, respectively. The authors attributed the more rapid glycaemic increase in fish receiving simple sugars than in those receiving starch to the necessity of starch hydrolysis before absorption and subsequent circulation, which is a finding consistent with the results observed in our starch-administered tambaqui.

In comparing the various carbohydrates used by Takahashi et al. (2018), glucose elicited the highest plasma glucose concentration (8.30 mmol/L), followed by starch (7.70 mmol/L) and fructose (5.71 mmol/L). In contrast, our investigation showed the highest glucose concentrations in tambaqui administered starch (6.20 mmol/L) and glucose (5.56 mmol/L), followed by sucrose (4.93 mmol/L), with the lowest peak observed in the fructose-treated group (4.03 mmol/L). In other fish species, such as Nile tilapia and piaú (*Leporinus elongatus*), the administration of a 1000 mg/kg BW fructose load did not induce a hyperglycaemic response over time. However, tambaqui in the same study did exhibit elevated plasma glucose levels (De Souza et al. 2021). The authors proposed that this differential response may be linked to the frugivorous dietary habits of tambaquis. Meanwhile, the other tested species showed reduced fructose absorption in the gastrointestinal tract, influencing plasma glucose concentrations. Given the natural consumption of fruits and seeds by tambaquis, it is plausible that this species exhibits metabolic and absorptive features that facilitate fructose utilization.

From a metabolic perspective, differences in intestinal absorption and hepatic handling may contribute to this pattern. Fructose uptake is known to occur via specific transporters, such as GLUT5 (Viegas et al. 2022), and its hepatic metabolism proceeds largely through insulin-independent pathways, entering glycolysis downstream of key regulatory steps. This metabolic routing may allow fructose to be efficiently utilized without generating marked elevations in circulating glucose. In the present study, the lower postprandial glycaemic peaks observed after fructose administration are consistent with such a mechanism. However, since transporter expression and metabolic fluxes were not directly assessed, this interpretation should be considered a physiological hypothesis requiring further investigation.

Shikata et al. (1994) conducted glucose, fructose, and galactose tolerance tests in common carp (*Cyprinus carpio*) and reported lower blood glucose peaks in fish administered fructose or galactose. They also observed undetectable baseline levels of fructose and galactose in the blood, with peak concentrations of 0.75 h post-administration. At 4 h post-administration, blood fructose levels returned to baseline. The authors concluded that fructose and galactose were not completely converted to glucose during intestinal absorption.

Dietary carbohydrates can upregulate the hepatic activities of PK and glucokinase (GK) enzymes in fish (Liu et al. 2018). In rainbow trout, dietary glucose induces an increase in hepatic GK expression, which has not been observed in fish fed fructose-containing diets (Panserat et al. 2001). However, our findings indicated an increase in hepatic GK activity in all sugar-administered tambaquis starting at 9 h post-administration. Hepatic GK enzyme activity is associated with dietary carbohydrate intake (Enes et al. 2009). According to Moon (2001), this enzyme plays a critical role in the normalisation of blood glucose levels. The observed increase in hepatic GK activity may explain the normalisation of blood glucose levels from 9 h post-sugar administration, underscoring the efficient capacity of this species to process all tested carbohydrate sources.

Efficient use of dietary carbohydrates for energy production typically necessitates the concomitant inhibition of hepatic gluconeogenesis. Panserat et al. (2000) reported that endogenous glucose synthesis was not effectively suppressed in rainbow trout fed high-carbohydrate diets. The impaired co-ordination between hepatic glycolysis and gluconeogenesis underscores a limitation in the efficient use of carbohydrates as a primary energy source in certain fish species (Panserat et al. 2000). Conversely, in our study, the observed increase in glucokinase (GK) activity was accompanied by the attenuation of the hepatic activity of the gluconeogenic enzyme fructose-1,6-bisphosphatase (FBPase), starting at 6 h in tambaqui receiving starch and at 9 h in those receiving glucose, fructose, or sucrose. This inverse relationship suggests a regulatory mechanism in tambaqui metabolism that facilitates efficient use of dietary carbohydrates.

The induction of hepatic lipogenesis serves as a crucial mechanism for the peripheral use of glucose, contributing to a reduction in circulating blood glucose levels in fish (Polakof et al. 2012). Although serum triglyceride concentrations did not show statistically significant temporal variations following sugar administration, an increase in hepatic lipogenesis based on the elevated hepatic activity of the FAS enzyme 6 h after starch administration and 9 h after glucose administration, which persisted until the end of the experimental period. This response indicates an active hepatic involvement in carbohydrate processing in tambaqui.

Although the observed increase in hepatic FAS activity in tambaqui-administered glucose and starch, those administered fructose and sucrose did not exhibit a similar trend. Differences in intestinal transport mechanisms and hepatic metabolic routing between glucose and fructose have been described in fish (Viegas et al. 2022). Fructose is absorbed through specific transporters and enters hepatic glycolysis downstream of key regulatory steps, which may influence its systemic glycaemic profile. The lower postprandial glycaemic peaks observed after fructose administration in the present study are consistent with a distinct metabolic handling of this monosaccharide. However, as intestinal absorption efficiency, portal transport, and microbiota-mediated metabolism were not directly assessed, these interpretations remain hypothetical. Previous studies in common carp (Shikata et al. 1994) reported that fructose did not promote lipogenesis, which aligns with the absence of FAS induction observed here. Further investigation is required to clarify the mechanisms underlying fructose absorption and metabolic partitioning in frugivorous fish species.

Although an increase in hepatic FAS activity was observed, no statistically significant changes were detected in serum triglycerides or total lipid concentrations. The transient variation in total serum lipids over time was not treatment-specific and should therefore be interpreted with caution. It is important to emphasize that the present study was designed as an acute carbohydrate tolerance experiment. Under short-term conditions, modulation of hepatic enzyme activities may precede detectable alterations in circulating lipid pools or tissue lipid deposition. Consequently, the observed increase in FAS activity should be interpreted as indicative of activation of lipogenic pathways rather than direct evidence of lipid accumulation or a meaningful contribution of lipogenesis to glucose clearance. Longer-term dietary studies including direct measurements of hepatic lipid content would be required to clarify these aspects.

Despite the marked glycaemic excursions and modulation of hepatic enzyme activities, hepatic glycogen content remained unchanged across treatments and sampling times. In acute carbohydrate tolerance tests, glycogen turnover may be rapid, and transient synthesis and degradation may not result in detectable net accumulation within the sampling window. Therefore, the absence of significant differences likely reflects stable net glycogen balance under short-term conditions rather than lack of glycogenic involvement.

Our findings indicate that juvenile tambaqui can physiologically respond to a 200 mg/100 g BW load of different carbohydrate sources, as reflected by the restoration of plasma glucose homeostasis and coordinated changes in hepatic carbohydrate-related enzyme activities. When compared with other fish species, the relatively rapid normalization of blood glucose suggests a regulated glycaemic response following carbohydrate administration. The observed modulation of hepatic glycolytic and lipogenic enzyme activities is consistent with hepatic participation in carbohydrate processing, although direct measurements of metabolic flux were not performed. Further studies incorporating measurements of transporters, hormonal regulation, and metabolic fluxes would be valuable to confirm and refine the mechanistic basis underlying these metabolic responses.

In the fructose-treated group, the absence of marked lipogenic enzyme induction combined with lower plasma glucose peaks suggests a distinct metabolic handling of this monosaccharide. While hepatic glycolytic and gluconeogenic enzyme activities indicate involvement of fructose-related metabolic pathways, the present data do not allow definitive conclusions regarding intestinal absorption efficiency or tissue-specific utilization. Further studies assessing transporters, hormonal regulation, and metabolic fluxes are required, particularly in frugivorous species such as tambaqui, in which fructose may represent a natural dietary component.

## Data Availability

The data supporting the conclusions of this study are available on a restricted basis in Zenodo at https://doi.org/10.5281/zenodo.17682858. Access will be granted upon request.
